# Cystic Kidney Diseases From the Adult Nephrologist’s Point of View

**DOI:** 10.3389/fped.2018.00065

**Published:** 2018-03-22

**Authors:** Roman-Ulrich Müller, Thomas Benzing

**Affiliations:** Department II of Internal Medicine and Center for Molecular Medicine Cologne, University of Cologne, Cologne, Germany

**Keywords:** polycystic kidney diseases, autosomal dominant polycystic kidney disease, autosomal-recessive polycystic kidney disease, tuberous sclerosis complex, von Hippel–Lindau disease, nephronophthisis, genetic kidney disease, Birt–Hogg–Dubé syndrome

## Abstract

Cystic kidney diseases affect patients of all age groups with the onset spanning from prenatal disease to late adulthood. Autosomal-dominant polycystic kidney disease (ADPKD) is by far the most common renal cystic disease. However, there are various cystic kidney diseases, the onset of which occurs at different times in life and depends on the type of the disease and the causative genes involved. When genetic kidney diseases are discussed in the adult setting this view is usually limited on autosomal-dominant kidney disease, the most frequent genetic disorder causing adult onset ESRD. Other diseases—such as autosomal-recessive polycystic kidney disease—are often being viewed as a disorder only important in pediatric nephrology. However, more recent data has revealed that, despite clear age peaks of onset for each disorder, all of them can also show highly variable phenotypes with classical adult onset genetic diseases being of importance in pediatrics and *vice versa*. Furthermore, the affected children need to be seen by adult nephrologists in the long term after transition, requiring knowledge on the underlying pediatric disease, potential extrarenal manifestations, and genetic counseling. Consequently, the view on these diseases should be widened on both ends. Close interaction between pediatric and adult nephrology is key to appropriate care of patients suffering from genetic kidney disease to profit from each other’s experience.

## Introduction

While pediatric nephrologists are rather experienced in managing patients with genetic kidney diseases, most adult nephrologists are faced with congenital disease less frequently (1, 2). This is mainly due to the reason that genetic disorders are the minority of causes of end-stage renal disease in the adult population whereas in children they are common. Autosomal-dominant polycystic kidney disease (ADPKD) reveals most of its burden in adulthood and has long been considered to be the only cystic kidney disease in adults. Other cystic kidney diseases—primarily nephronophthisis (NPH) and autosomal-recessive polycystic kidney disease (ARPKD)—start much earlier and are entities that have primarily been studied and treated by pediatric nephrologists. However, with the advent of the first targeted treatment strategies—such as tolvaptan in ADPKD patients—the question arises whether early detection of disease and an early commencement of a potential therapy may be beneficial.

Genetic causes of kidney disease are on the brink to become much more of a visible problem in adult nephrology. This is not limited to cystic kidney diseases, but also true for other disorders, such as the MCD-FSGS spectrum. On the one hand, an increasing number of children suffering from kidney disease reach adulthood raising the question of transition and continued care in the adult nephrology setting. On the other hand, the advent of novel sequencing technologies has paved the way for elucidating more complex genetic factors in kidney disease and will allow for novel associations between the genetic background and pathogenic aspects. Clearly, chronic kidney disease in general in adulthood has an important genetic component. However, many of the genetic alterations are not simply monogenetic causes, but must rather be considered as complex genetic diseases. Consequently, knowledge on genetic diseases of the kidney will play an increasingly important role in adult nephrology and profit from a close interaction with colleagues from pediatric nephrology. Here, we will use the example of cystic kidney diseases—and ADPKD specifically—to point out the most important aspects and advances in the care of adult patients.

## Cystic Kidney Disease—A Ciliopathy

The introduction of next-generation sequencing techniques in the last decade—starting in basic research and being currently introduced in clinical diagnostics—made the large-scale analysis of potential disease-causing mutations in patients with cystic kidney diseases feasible (3–6). This has led to the identification of numerous novel disease-causing genes enabling the discovery of more than 100 genes to be involved in cystic kidney diseases (7). Nonetheless, a significant proportion of cases remains unsolved which will require further research in well-characterized families and cohorts (3, 5). Despite this enormous genetic complexity of cystic kidney diseases, they are unified by a single pathophysiological concept. Nearly all protein products of disease-causing genes identified so far have been linked to the generation or function of the primary cilium—a membranous protrusion of the apical cell membrane of close to all cell types that is supported by a microtubular skeleton (7). Primary cilia may function as chemo- and mechanoreceptors and ciliary dysfunction which initiates numerous signaling aberrations in epithelial kidney cells—like increased cAMP generation or mTOR-signaling—that are crucial to cyst formation (8). This has led to the definition of the cystic disease complex as a ciliopathy. The fact that pretty much all tissues in the human body contain ciliated cells explains why ciliary defects can be the basis to complex syndromes affecting different organs—e.g., the combination of cystic kidneys with *situs inversus* and retinitis pigmentosa observed in several NPH-associated syndromes (9).

## ADPKD—Epidemiology

Autosomal-dominant polycystic kidney disease is the most common entity among cystic kidney diseases (10). Its lifetime morbid risk is estimated to be around 1:1,000 while point prevalence—which rather reflects clinically relevant disease—has recently been shown to be approximately 1:2,500 (11–14). The frequency of ADPKD is thus comparable to much more commonly known disorders, such as cystic fibrosis or multiple sclerosis. Consequently, it is also the most frequent genetic cause of end-stage renal disease in adults with a prevalence of 5–10% in the dialysis population (15). However, a decline in renal function is rarely observed in childhood leading to ADPKD being primarily viewed as a disease of adult patients. Nonetheless, with the advent of novel targeted therapeutic strategies—e.g., tolvaptan and somatostatin-analogs—and seeing that a subpopulation of children with ADPKD suffer from clinically meaningful hypertension (16), ADPKD has gained increasing attention among pediatric nephrologists—a fact that is reflected by the initiation of a pediatric ADPKD registry in 2017 (www.adpedkd.org) that has successfully started recruiting patients. Nonetheless, the predominant time at which ADPKD gets manifest is above the age of 30 years and ESRD is reached at an average age of 50–60 years (17). Consequently, while there is no clear consensus of how to care for children of affected parents, the period in life during which interventions are necessary is primarily adulthood for now. Obviously, this may change in the future, e.g., with upcoming clinical trials on drugs such as the V2 receptor antagonist tolvaptan in pediatric cohorts (18).

## Differential Diagnosis of ADPKD

Due to its typical clinical manifestation, the diagnosis of ADPKD can be made solely on clinical grounds in the vast majority of affected patients. This is primarily based on three considerations: (1) morphology of the kidneys, (2) extrarenal manifestations, and (3) mode of inheritance.

Using these criteria ADPKD can—in most cases—clearly be separated from the rarer causes of cystic kidney diseases in adulthood. This is very important, since—even though ADPKD is by far the most common diagnosis—distinguishing other disorders, including autosomal-dominant tubulointerstitial disease (ADTKD), NPH, and ARPKD and a clear separation from simple renal cysts is crucial to make the correct diagnosis. This has become even more important with targeted therapies getting available for particular disorders.

### Kidney Morphology

With the morphology of the kidneys being the key to diagnosing or excluding ADPKD in individuals at risk, imaging modalities are highly important screening tools. As to this point, several different key factors have to be taken into consideration: (1) kidney size, (2) number of cysts, (3) unilateral/bilateral disease, and (4) distribution of the cysts.

**Figure 1:**
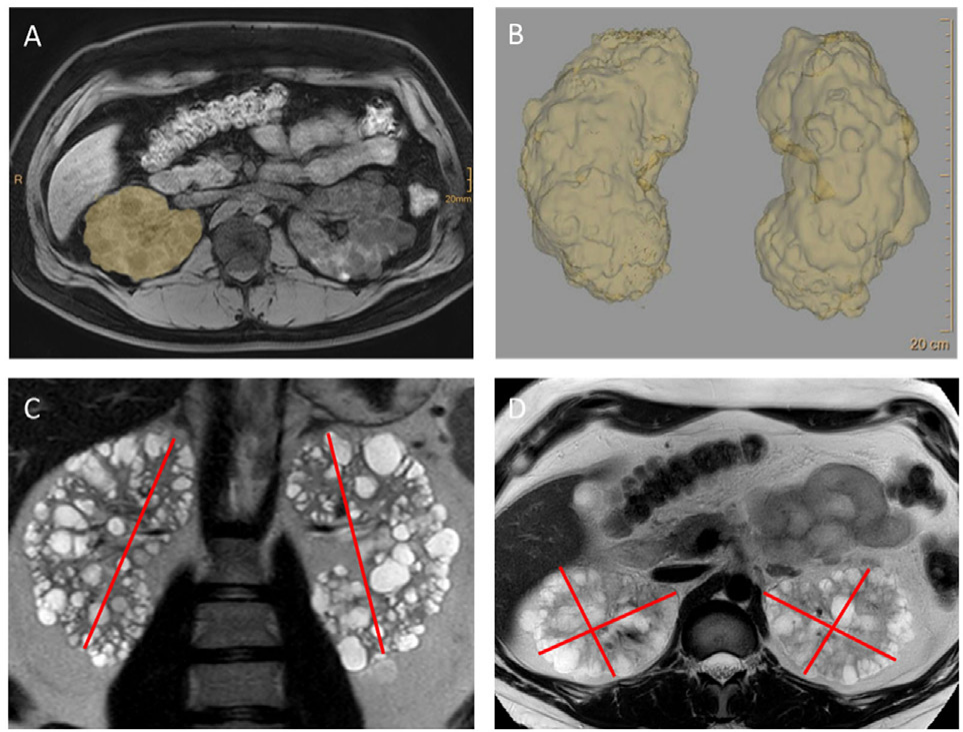
MRI imaging and volumetry of kidneys in autosomal-dominant polycystic kidney disease (ADPKD). These images reveal the classical features of ADPKD: strongly enlarged kidneys showing a ubiquitous distribution of cysts throughout the parenchyma. Kidney volume—an important prognostic feature now used in algorithm for making treatment decisions—can be obtained by planimetry **(A,B)** as done in the clinical trials. However, for everyday clinical decisions volumetry based on measuring the axes and using the ellipsoid formula has been shown to be sufficient (19) **(C,D)**. From Müller et al. (27) (images kindly provided by Dr. Thorsten Persigehl, Institute of Radiology, University of Cologne).

(1) Autosomal-dominant polycystic kidney disease generally goes along with enlarged kidneys. Since the disease is gradually progressing during lifetime, size has to be considered in an age-dependent fashion. The correlation of age-adjusted kidney size with disease progression allows for using the information obtained by imaging to—beyond making a diagnosis—help guiding treatment decisions using, e.g., MRI volumetry (19) (Figure 1). Large polycystic kidneys are also found in ARPKD as well as the much less common tumor-associated syndromes (tuberous sclerosis, Von Hippel–Lindau disease, Birt–Hogg–Dubé syndrome) and *HNF1b*-associated kidney disease (20–22). In contrast to this, diseases of the NPH complex are characterized by small (to normal) sized kidneys. (2) Ravine et al. were the first to come up with a classification based on cyst numbers that allowed for diagnosing ADPKD in individuals with a positive family history using ultrasonography more than 20 years ago (23). Since then this approach has been revised twice in order to account for the advances in imaging technologies that strongly increased sensitivity of cyst detection accompanied with decreasing positive predictive values (24, 25). York Pei and colleagues validated novel criteria in a well-designed study in 2015 that now allows for clear detection of ADPKD by ultrasound from the age of 30 onwards. Interestingly, all affected individuals showed more than ten cysts when measured by MRI clearly distinguishing them from unaffected individuals. This held true already from the age of 16 years providing data that imaging-based exclusion of disease may be possible starting in adolescence (25). One caveat regarding the diagnosis of ADPKD based on cyst number is the fact that these approaches have only been validated in patients showing a positive family history. (3) However, there is still more conclusions that can be drawn from kidney morphology in cystic kidney diseases. First, based on the fact that these are genetic diseases that go along with the same genetic alteration in all kidney cells, bilateral disease is to be expected. There is few and very rare exceptions to this rule due to potential genetic somatic mosaicism (26). (4) In ADPKD, the bilateral cysts are generally distributed ubiquitously throughout the parenchyma, since cyst formation occurs along the entire nephron (Figure 1). This leads to an entirely different pattern compared to NPH, where the cysts are strictly localized at the corticomedullary junction. In ARPKD, cyst formation occurs primarily in the collecting duct, however, the imaging findings can be similar to ADPKD. The same holds true for the tumor syndromes mentioned above and *HNF1b*-associated disease that can all phenocopy ADPKD as to kidney morphology. Here, apart from the fact that—due to its frequency—ADPKD is clearly the most likely diagnosis, extrarenal findings help to distinguish the different entities.

### Renal Manifestations Beyond Cysts

As to the kidneys themselves, ADPKD is not only characterized by renal cysts but also includes kidney stones, flank pain, cyst infections, and macrohematuria. While these manifestations are common in adult patients and may lead to the diagnosis—especially in cases with a negative family history—they are rare in childhood. Importantly, early onset of these symptoms may indicate rapid progression of disease as pointed out by the data from the French GENKYST cohort (PROPKD score, see section on “Targeted therapies”) (28).

### Extrarenal Manifestations

As explained above cystic kidney diseases are one of the best characterized ciliopathies. Knowing that cilia are present on nearly all cells of the human body and are expected to play important roles in cellular polarity signaling and cell proliferation it is not surprising that the clinical manifestation of a ciliopathy is normally not limited to one organ, but causes a multitude of syndromic clinical pictures. Knowledge of the extrarenal manifestations is crucial to patient care, since they do not only explain symptoms but may also require specific interventions/diagnostic strategies—e.g., in the case of intracranial aneurysms in ADPKD. As to diagnosing a specific cystic kidney disease these manifestations are very helpful, especially in cases where the decision is not clear based on kidney morphology. Hence, a complete workup of these aspects by patient history and clinical examination is essential when seeing PKD patients.

### Extrarenal Manifestations in ADPKD

The most common extrarenal finding in ADPKD patients is extrarenal cysts with the majority of patients also showing liver cysts (and less frequently in other organs, such as the seminal vesicles, pancreas, or spleen). Furthermore, diverticulosis is a common manifestation. As to cardiac manifestations cardiac valve abnormalities—primarily mitral valve prolaps and insufficiency—are the most common findings, a fact that makes echocardiography a useful tool in the workup of these patients. Abdominal and inguinal hernias are also associated with ADPKD and are probably a consequence of both an altered strength of the abdominal wall and the increased intra-abdominal pressure due to enlarged kidneys and liver. An aspect that may be especially worrisome for ADPKD patients is the increased occurrence of intracranial aneurysms. Here, a tailored screening strategy in high-risk patients (e.g., those with a positive family history) as well as an interdisciplinary workup (neurosurgeons, neuroradiologists, nephrologists) are crucial—especially when taking into account that interventions to close these aneurysms can also lead to significant morbidity and mortality (29, 30). A more complete workup of potential extrarenal associations with ADPKD and their management can be found in the recent literature (10, 31, 32).

### Extrarenal Manifestations of Tumor Syndromes Associated With Polycystic Kidneys

In contrast to ADPKD, the tumor syndromes—associated with polycystic kidneys that often resemble ADPKD—tuberous sclerosis, BHD syndrome, and VHL syndrome—all go along with a significantly increased risk of kidney cancer (clear-cell renal cell carcinoma in VHL and TSC; chromophobe renal cell carcinoma in BHD). Furthermore, all of these syndromes have characteristic extrarenal manifestations—TSC: e.g., giant-cell astrocytomas and other CNS tumors, renal angiomyolipomas, pulmonary lymphangioleiomyomatosis, cardiac rhabdomyomas, and multiple dermatological signs (facial angiofibromas, periungual fibromas, ash leaf spots, Shagreen patches, etc.); BHD: recurrent pneumothoraces and fibrofolliculomas; VHL: e.g., CNS/retinal hemangioblastomas, pancreatic neuroendocrine tumors, and pheochromocytoma (33). All of these can and should be used for differential diagnosis. While VHL and BHD patients often do not show any symptoms before late adolescence or adulthood, TSC—especially due to the CNS and cardiac manifestations—is frequently already highly symptomatic in early childhood. Nonetheless, there are a considerable proportion of TSC patients that are not diagnosed before adulthood and present with an oligosymptomatic course that, e.g., is mainly characterized by multiple renal angiomyolipomas.

### Extrarenal Manifestations in ARPKD and HNF1B-Associated Kidney Disease

Another disease that can phenocopy the renal phenotype of ADPKD patients is ARPKD. However, the spectrum of extrarenal manifestations differs between these two entities. While in childhood pulmonary hypoplasia is one of the most dramatic findings, patients reaching adulthood without having been diagnosed do generally not show pulmonary symptoms. However, ARPKD has an obligatory association with liver fibrosis (in combination with dilated bile ducts—Caroli syndrome). Consequently, these patients need a workup, including liver imaging (sonography, fibroscan) and gastroscopy (screening for varices). Combined kidney-liver transplantation always needs to be considered when reaching end-stage renal disease (34).

HNF1b is a key transcription factor influencing numerous renal and extrarenal disease genes. Consequently, its mutation can lead to a range of symptoms. As to extrarenal manifestation-associated disorders, which include elevated liver enzymes, MODY diabetes mellitus, pancreatic insufficiency, hypomagnesemia, hyperparathyroidism, gout, and mental retardation (22).

### Extrarenal Manifestations in Diseases of the NPH Complex

Even though diseases of the NPH complex are much less common in the adult population knowledge about their characteristics is important for identifying individuals affected by non-ADPKD cystic kidney diseases. Due to the nature of these disorders as a ciliopathy a wide range of affected tissues and symptoms are possible. In the following, we will summarize the most common examples that may sometimes not be diagnosed before reaching adulthood (9). Importantly, the attribution of a disorder to the NPH complex is—due to genetic complexity—not primarily guided by genetic diagnostics, but by the clinical characterization of the syndromic pattern of manifestations. The most frequent extrarenal finding in NPH patients is retinitis pigmentosa, which is characterized by night blindness followed by tunnel vision and eventually blindness. The combination of NPH and retinitis pigmentosa is classified as Senior–Løken syndrome. Furthermore—based on the role of cilia at the primary node during embryonal determination of laterality—many patients with NPH-associated syndromes show *situs inversus* as a characteristic finding. Another entity to be specifically named here is Bardet–Biedl syndrome which is characterized by polydactyly, juvenile obesity (often accompanied by diabetes mellitus), mental retardation of different degrees, retinitis pigmentosa, anosmia, and hypogonadism. Interestingly, Bardet–Biedl patients often show large kidneys at birth. While kidney size in the majority returns to normal by the age of 1–2 years, renal phenotypes that resemble ADPKD have been described in adult patients. Most other diseases of the NPH complex are nearly exclusively diagnosed in early childhood, such as Joubert syndrome (characterized by cerebellar vermis hypoplasia) or result in perinatal lethality as for Meckel–Gruber syndrome (20).

### The Role of Genetic Diagnostics

**Figure 2:**
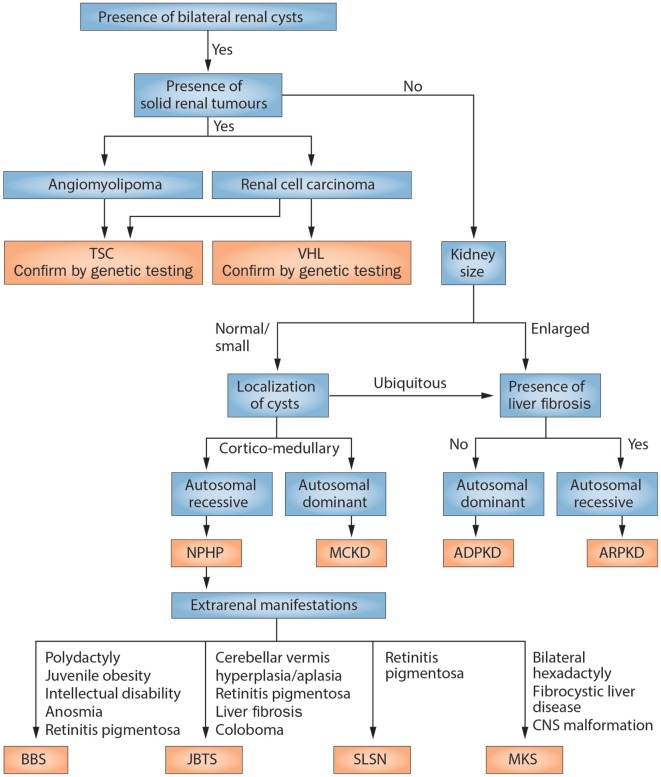
Scheme of the diagnostic algorithm for polycystic kidney diseases combining clinical judgment with imaging modalities and molecular genetics. From Kurschat et al. (20). NPHP, nephronophthisis; MCKD, medullary cystic kidney disease (better to be termed now: autosomal-dominant tubulointerstitial disease); BBS, Bardet–Biedl syndrome; JBTS, Joubert syndrome; SLSN, Senior–Loken syndrome; MKS, Meckel–Gruber syndrome.

Using the clinical criteria mentioned above, the diagnosis can be made correctly in the vast majority of patients without requiring a molecular genetics workup (20, 25, 35). In NPH, there is only very limited phenotype–genotype correlation which makes targeted genetics difficult. However, since *NPHP1* is the most commonly affected gene, testing for alterations in *NPHP1* may be of diagnostic utility (36, 37). This may change in the future with always cheaper and easier access to next-generation sequencing panels. ADPKD is—in patients with a positive family history—diagnosed almost exclusively on clinical grounds. However, molecular genetics may play a major important role in these patients for predicting disease progression (which may be a piece of the puzzle for making therapeutic decisions) in the future (28). The tumor syndromes on the other hand require different considerations when discussing molecular genetics. Here, a clear diagnosis based on the mutation is essential to distinguish these entities from ADPKD which then allows for the design of screening strategies for individual patients and prognostic testing in family members. An overview of a diagnostic strategy in cystic kidney diseases including clinical and genetic considerations is illustrated in (Figure 2). Importantly, current clinical diagnostic algorithms may require adaptation in the future due to novel culprit genes and associated disease entities identified—e.g., *GANAB* recently described as a new gene in ADPKD (5). Obviously, diseases not expected in adulthood—e.g., ARPKD and rare disorders that go along with variable clinical manifestations like HNF1b-associated kidney disease—may also require a molecular genetic diagnosis.

## Clinical Trials to Prevent Disease Progression of Cystic Kidney Disease

### Supportive Measures

Knowledge of the various manifestations is key to the therapy of cystic kidney diseases in general. ESRD can obviously be addressed by renal transplantation in all entities (with a combined liver transplantation to be considered in ARPKD patients). Yet, data from randomized trials as well as a targeted therapy are only available for ADPKD. Until the year 2015 merely supportive measures were available. The most important recommendations to all of the affected individuals are summarized in Table 1, however, the degree of evidence is limited for some of these recommendations. As an example, sufficient fluid intake is mainly based on pathophysiological considerations regarding ADH-suppression (38–40). Here, a recently initiated randomized trial will hopefully clarify the impact of fluid intake (41). On the contrary, there is solid data regarding blood pressure control which was recently emphasized again by the randomized HALT-PKD trials (42). Study arm A showed that lowering blood pressure to 95/60–110/70 mmHg compared to 120/70–130/80 mmHg using an ACE-inhibitor or an AT1-blocker leads to a significant decrease in the kidney growth rate (42) in young patients with preserved kidney function (15–49 years of age, CKD stages G1 and 2). Dual RAAS-blockade once again did not show any additional benefit (43). Interestingly, a *post hoc* analysis of the same trial confirmed that salt restriction should be part of the general management of ADPKD patients (44).

**Table 1:** Supportive measures in autosomal-dominant polycystic kidney disease (ADPKD).

Supportive measures in ADPKD
Blood pressure control (42)
Limiting NaCl intake to <5–7 g/day (44)
Sufficient fluid intake (>3 L/day) (38–41)
Avoid estrogen intake (which stimulates liver growth) (45–47)
Healthy diet (e.g., Mediterranean diet) (48, 49)

*While strict blood pressure control is based upon the results from an RCT (HALT-PKD study arm A), the strongest data regarding limiting salt intake specifically in ADPKD relies on a *post hoc* analysis of HALT-PKD. The effect of estrogens was observed both in pregnant women and more specifically in a small cohort regarding postmenopausal substitution. As to healthy diets, trial results regarding cardiovascular endpoints—the risk of which is increased in ADPKD—can be extrapolated to this cohort. Sufficient fluid intake inhibits ADH secretion, however, its impact on disease progression still needs to be confirmed*.

### Targeted Therapies—Tolvaptan

The TEMPO 3:4 trial which was published in 2012 entirely changed the approach to ADPKD, since it led to the approval of the very first targeted therapy in Europe. TEMPO 3:4 showed—in a double-blind randomized design—that vasopressin receptor (V2R) inhibition could significantly slow down the rate of kidney growth by close to 50% (50). Even more importantly, eGFR loss was decreased by about 26%, an effect size that is comparable to the large trials on RAAS-blockade in diabetic nephropathy. This effect has recently been confirmed by the REPRISE trial that showed tolvaptan to be effective not only in early-stage ADPKD but also in patients having reached early CKD stage G4 (51). As expected V2R-blockade goes along with significant polyuria. Nonetheless, close to 80% of patients enrolled did continue through 3 years of the trial; a finding that is confirmed by recent real-world experiences (52). Furthermore, even though rare and reversible, liver toxicity is possible and requires regular screening of liver enzymes. In order for ADPKD patients to benefit from this therapy, careful selection of patients in whom the treatment is started is of utmost importance. Individuals that will not reach ESRD during lifetime are not suitable candidates for such an approach making only rapid progressors candidates for this therapy. Consequently, several publications have been published in the past 2 years trying to give guidance to nephrologists as to patient selection (27, 53). Past data on eGFR loss is the best indicator of rapid disease progression, yet, these data are not always available and do not help in CKD stage G1. Consequently, a row of predictors of disease progression in ADPKD—including models using total kidney volume (“Mayo classification”) (19), mutation status, and clinical symptoms (“PROPKD Score”) (28)—have been validated that can help in treatment decisions and patient counseling. While ultrasonography is usually sufficient to make a diagnosis of ADPKD, MRI volumetry has proven very helpful to obtain more precise kidney volumes as the basis to treatment decisions (Figure 1). A more detailed summary of these criteria can be found in the recent literature (27, 53).

### Targeted Therapies Beyond Tolvaptan

Even though tolvaptan is a major step forward in the care of ADPKD patients it only slows down disease progression and will not prevent reaching ESRD in the vast majority of patients. Consequently, additional strategies targeting others of the multiple dysregulated cellular signal transduction pathways are necessary (54). One promising target was the mTOR-signaling pathway based on data from mouse models (55, 56). However, two clinical trials did unfortunately not show any benefit in the patient setting (57, 58). The use of somatostatin-analogs targets the same intracellular secondary messenger—cAMP—as tolvaptan. A small trial has shown a potential benefit in ADPKD patients (59). Currently, two phase 3 trials—the LIPS trial in France and the DIPAK1 trial in the Netherlands—are examining this approach in larger cohorts. Somatostatin-analogs would have the advantage to also influence liver disease in ADPKD, while an impact on the hepatic involvement has by now not been shown in the case of tolvaptan. Interestingly, with the use of pravastatin in ADPKD, a double-blind randomized trial in children and young adults has added another potential target (60). Even though a recent *post hoc* analysis of the HALT-PKD trials could not confirm a disease-modifying effect of statin therapy in ADPKD, final clarification of the potential of these drugs will need a larger prospective clinical trial (61). With the overactivation of several kinases in ADPKD, re-purposing of kinase inhibitors has gained interest in the past years. In this line, a phase 2 trial examining the potential of bosutinib—a src/bcr-abl tyrosine kinase inhibitor—has recently been published. In this 2-year trial, bosutinib was able to slow down kidney growth. However, there was no effect on eGFR loss and close to 50% of the patients discontinued the initial treatment period, primarily due to treatment-associated adverse events (62). Consequently, more efforts are necessary to continue translating basic research in ADPKD to clinically meaningful therapies. Apart from the identification of novel treatment targets and their translation into clinical trials—e.g., as for CDC25 inhibition using vitamin K3, treatment with niacinamide, re-purposing of metformin, dietary interventions, or reversal of the Warburg effect (63–67)—combination therapies could also help in improving the effects and reducing side effects of pharmacological therapies in ADPKD in the future (68, 69). Furthermore, as to tolvaptan, a trial that started recruitment in the past year is currently examining V2R-inhibition as a therapeutic principle in a pediatric cohort. Even though clinical disease onset in ADPKD does primarily occur in adulthood, patients with clear indicators of very rapid disease progression could profit from an early start of therapy making this trial an interesting endeavor.

## Conclusion

There has been an enormous breakthrough in the understanding of polycystic kidney diseases both in the adult as well as in the pediatric population. A large number of underlying gene defects have been identified and pathogenic pathways characterized. In ADPKD this resulted in the development of the first available treatment strategies and the approval of tolvaptan to prevent progression of the disorder. Even though ADPKD is primarily a disease of adulthood and ADPKD patients are thus mainly seen by adult nephrologists, a close interaction between pediatric and adult nephrology will be of benefit to patients and their families. Not only do genetic diseases always involve more than one individual. Unusual courses can also lead to onset of classical diseases of adulthood (e.g., ADPKD) in children and of classical pediatric diseases (e.g., ARPKD and NPH complex) in adult patients. Adult nephrologists can benefit from the wide experience of pediatric disciplines regarding genetic diseases on the one hand. On the other hand, this interaction helps in combining the knowledge on childhood interventions with outcome in adulthood and allows to learn from each other’s experiences in the treatment of polycystic kidney diseases.

## Author Contributions

R-UM and TB wrote the manuscript. R-UM designed the figures.

## Conflict of Interest Statement

R-UM and TB have received personal fees for participation in advisory boards and as an expert speaker from Otsuka Pharmaceutical. The Department II of Internal Medicine has received research funding from Otsuka Pharmaceutical.
